# Lectin-based analysis of fucosylated glycoproteins of human skim milk during 47 days of lactation

**DOI:** 10.1007/s10719-015-9615-5

**Published:** 2015-08-30

**Authors:** Jolanta Lis-Kuberka, Iwona Kątnik-Prastowska, Marta Berghausen-Mazur, Magdalena Orczyk-Pawiłowicz

**Affiliations:** Department of Chemistry and Immunochemistry, Wrocław Medical University, Bujwida 44a, 50-345 Wrocław, Poland; 1st Department and Clinic of Gynaecology and Obstetrics, Wrocław Medical University, T. Chałubińskiego 3, 50-368 Wrocław, Poland

**Keywords:** Human milk glycoproteins, Fucosylated glycotopes, Lactation, Lectins, Fucose

## Abstract

Glycoproteins of human milk are multifunctional molecules, and their fucosylated variants are potentially active molecules in immunological events ensuring breastfed infants optimal development and protection against infection diseases. The expression of fucosylated glycotopes may correspond to milk maturation stages. The relative amounts of fucosylated glycotopes of human skim milk glycoproteins over the course of lactation from the 2^nd^ day to the 47^th^ day were analyzed in colostrums, transitional and mature milk samples of 43 healthy mothers by lectin-blotting using α1-2-, α1-6-, and α1-3-fucose specific biotinylated *Ulex europaeus* (UEA), *Lens culinaris* (LCA), and *Lotus tetragonolobus* (LTA) lectins, respectively. The reactivities of UEA and LCA with the milk glycoproteins showed the highest expression of α1-2- and α1-6-fucosylated glycotopes on colostrum glycoproteins. The level of UEA-reactive glycoproteins from the beginning of lactation to the 14^th^ day was high and relatively stable in contrast to LCA-reactive glycoproteins, the level of which significantly decreased from 2–3 to 7–8 days then remained almost unchanged until the 12^th^–14^th^ days. Next, during the progression of lactation the reactivities with both lectins declined significantly. Eighty percent of α1-2- and/or α1-6-fucosylated glycoproteins showed a high negative correlation with milk maturation. In contrast, most of the analyzed milk glycoproteins were not recognized or weakly recognized by LTA and remained at a low unchanged level over lactation. Only a 30-kDa milk glycoprotein was evidently LTA-reactive, showing a negative correlation with milk maturation. The gradual decline of high expression of α1-2- and α1-6-, but not α1-3-, fucoses on human milk glycoproteins of healthy mothers over lactation was associated with milk maturation.

## Introduction

Human milk of a healthy mother supports adequate growth and development of infants, covering nutrient requirements during the first 6 months of life [1–3]. Breast-feeding is connected with neurodevelopmental advantages [1, 2] and reduces the risk of acute and chronic diseases in the developing infant [4–7]. These properties are dependent on bioactive compounds, including fucosylated and sialylated glycoconjugates, which constitute a large part of the human milk and include free oligosaccharides (HMOs), glycoproteins, glycolipids and glycosaminoglycans [8–11]. Among milk glycoconjugates, the highly fucosylated and sialylated glycans take part in the protection of the newborn and infant from bacterial and viral infections [4, 10, 12, 13], and according to Royle *et al*. [14] they are among the components of the innate immune system.

The fucose on N- and O-glycans of glycoproteins occurs as a terminal monosaccharide and can be linked by α1-3/α1-4- glycosidic bonds to the subterminal GlcNAc or by an α1-2- bond to Gal of antennae [11, 15–17]. Fucose can also be attached by α1-6- linkage to GlcNAc of the core structure of N-glycans and is referred to as the core fucose or innermost fucose [11, 15–17]. The fucosylated human milk glycoproteins, similar to fucosylated plasma glycoproteins, can take part in biological recognition reactions, and their expression is reported to be bound with pathophysiological status. α1-6-linked fucose controls many biological reactions such as cell-cell signaling and adhesion, stimulates cell growth and differentiation and modulates IgG1-type antibody-dependent cellular cytotoxicity [15, 18]. Outer arm α1-2- and α1-3-linked fucoses are a part of the Lewis family glycotopes expressed on N- and O-glycans of glycoproteins [15–17] and can be recognized and bound by the lectin- receptors of bacteria and/or viruses, and in that way by blocking of lectin- receptors of pathogens prevent their adhesion to the host epithelial cells and prevent colonization and invasion of mucosa [5, 7, 11, 19–21]. On the other hand, α1-3-linked fucose is a part of the Lewis^x^ and sialo-Lewis^x^ antigens, which are ligands for L-, E-, and P-selectins [15, 22]. According to Bernardi *et al*. [21], the multivalent presentation of mostly larger and branched glycoprotein glycans could provide a better mechanism of pathogen capture than the simple and relatively small structures of HMOs. The presence or absence of α1-2- fucosylated glycotopes on cellular and soluble glycoconjugates of secretions (saliva, milk, semen) is genetically determined as secretor status. The milk of women who are secretors contains a higher amount of fucosylated glycoconjugates, which are closely connected with anti-microbial properties of human milk [reviewed in [7, 11, 20, 23]]. It has been shown that fucosylated glycoproteins of human milk inhibit binding of *Campylobacter jejuni* [24], enteropathogenic *Escherichia coli* (EPEC) [25], *Helicobacter pylori* [26], *Salmonella enterica* serovar Typhimurium and Heidelberg [6, 27], Noroviruses [28] and human immunodeficiency virus (HIV) to the host cells [29, 30]. It was also reported that α1-2-linked fucose has a potential to modulate growth, communication, and regeneration of neurons and can take part in forming long-term memory [31, 32]. Moreover, fucosylated glycans of milk glycoconjugates can be degraded by bacterial fucosidases produced in particular by *Bifidobacterium* species, which in this way can gain access to the energetic content of milk and predominance in the intestinal microbiota in the first year of infant life [11, 33].

The analysis of glycoprotein pattern in a human milk sample is complex, mainly because of different amounts of individual glycoproteins in the total pool and alterations in their concentration over lactation. Methods such as high performance liquid chromatography (HPLC) and mass spectrometry (MS) have been used for such determinations, but they are limited to selected stages of lactation as well as to a low number of samples analyzed [34]. Nwosu and coworkers [34] reported high fucosylation (75 %) and lower sialylation (57 %) of human milk N-glycans, but the analysis was limited to mature milk only. On the other hand, the detailed analysis of human milk oligosaccharides reported by De Leoz *et al*. [23] revealed that 63.5 % of them are fucosylated. To date, the knowledge concerning lactation-stage related glycovariants in human milk glycoproteome is limited to the major glycoproteins (S-IgA [14, 35], lactoferrin [6, 36], mucins [37]) and is mainly restricted to the selected weeks of lactation. A semi-quantitative overview of glycoprotein expression and changes in glycosylation profile of selected human milk glycoproteins during lactation was published by Froehlich [36]. Moreover, the sialylation and fucosylation of α-1-acid glycoprotein [38], fibronectin [39] and bile-salt-stimulated lipase (BSSL) [40] are lactation-stage related. Additionally, Gustafsson and coworkers [41] have shown that expression of fucosylated and sialylated glycotopes on human milk glycoproteins is the most complex one, and similar expression to that was observed for pig and horse but not for bovine milk proteins.

The aim of this study was to investigate the expression of α1-2-, α1-3- and α1-6- fucosylated glycotopes on human milk glycoproteins during the progression of milk maturation over 2–47 days of a healthy mother’s lactation. The fucosylated glycotopes on human milk glycoproteins were analyzed semi-quantitatively by lectin-blotting using lectins able to recognize and differentiate the type of glycosidic linkage to the oligosaccharide backbone and specific to α1-2-linked (UEA: *Ulex europaeus* lectin), α1-6-linked (LCA: *Lens culinaris* lectin), and α1-3-linked (LTA: *Lotus tetragonolobus* lectin) fucoses. The application of lectins has been especially helpful in obtaining information about the expression of biologically active glycotopes in their conformational native form, exposed and ready to react with natural receptors, such as endogenous selectins and bacterial lectins. Moreover, a lectin-based test allowed for simultaneous analysis of many milk glycoproteins and avoided a labor- and cost intensive procedure of isolation of individual glycoproteins. The analyses of milk samples obtained from healthy mothers were performed in the groups of colostrum of days 2, 3, and 4–5, transitional milk days 7–8, 10, and 12–14, and mature milk days 15–17, 30–35, and 39–47. The analysis of obtained data allowed us to observe the types of fucosylation changes associated with the milk maturation process.

## Materials and methods

### Participants

Samples of milk (*n* = 43) were obtained from healthy lactating women (from 21 to 35 years old) receiving regular perinatal care at the 1^st^ Department of Gynecology and Obstetrics at Wroclaw Medical University, Wroclaw, Poland. For inclusion in the study, participants had to have a good state of health and normal uncomplicated pregnancy. Women who used tobacco products, illicit drugs, or alcohol or with abnormal lactation (*e.g*., mastitis) or were pregnant with multiple fetuses were excluded. All mothers who agreed to give their milk for the biochemical research were acquainted with the protocol approved by the Ethics Committee at Wroclaw Medical University (number KB-30/2013). Informed consent was obtained from all participants.

### Sample collection and preparation

Samples of human milk from 2 to 47 days of lactation were collected by a trained nurse from the breast by manual expression at the end of nursing (hindmilk) by complete breast emptying, once per day, at the same time (8:00–10:00 a.m.). All milk samples were frozen in plastic containers and stored immediately at – 20 °C until analysis. Skim milk (aqueous phase) was prepared by centrifugation at 3,500 g at 4 °C for 35 min, after which the fat layer and cells were removed. The samples of skim human milk were stored at - 20 °C, and before the analysis, the aliquots of the human skim milk samples were kept for 1 h at room temperature.

For analysis of fucosylation of milk glycoproteins, the samples were collected only from mothers who have secretor status with Se+/Le + fenotype (information obtained from previous studies [38, 39]). To minimize the impact of individual differences among the mothers, the selected milk samples from the same period of the successive stages of lactation were pooled before analysis by mixing an equal volume of 200 μL of an individual skim milk sample. The following groups were formed:

1. colostrum (Day 2 of lactation; n = 2),
2. colostrum (Day 3 of lactation; n = 5),
3. colostrum (Day 4–5 of lactation; n = 7),
4. transitional milk (Day 7–8 of lactation; n = 5),
5. transitional milk (Day 10 of lactation; n = 4),
6. transitional milk (Day 12–14 of lactation; n = 5),
7. mature milk (Day 15–17 of lactation; n = 5),
8. mature milk (Day 30–35 of lactation; n = 5),
9. mature milk (Day 39–47 of lactation; n = 5).

## Methods

### Determination of protein concentration

The total protein concentration in human skim milk pooled samples was determined by bicinchoninic methods with the Bicinchoninic Acid Protein Assay Kit (Sigma, St. Louis, MO, USA) and bovine albumin as a standard.

For the analysis, 0.5 μl of human pooled skim milk, 24.5 μl of 0.9 % NaCl and 200 μl of freshly prepared bicinchoninic acid working reagent (solution of bicinchoninic acid and copper (II) sulfate in the ratio 1:50, respectively) were added to the same well of microtiter plates, and were incubated at 37.5 °C for 30 min. The absorbance was measured in a Stat Fax 2100 Microplate Reader (Awareness Technology Inc., Palm City, Florida, USA) at 560 nm. All samples were analyzed in duplicate.

### SDS- electrophoresis

The skim milk pooled sample containing 30 μg of protein was denatured at 100 °C for 5 min in the presence of 5 % β-mercaptoethanol, 10 % glycerol, 2 % SDS, 1 M Tris–HCl pH 6.8, and loaded to SDS-PAGE in a 7.5 % gel, according to Laemmli [42]. The electrophoresis was carried out in 0.1 % SDS buffer (25 mM Tris, 0.192 M glycine, 0.1 % SDS, pH 8.3) at 200 V for 50 min in the electrophoresis apparatus (Bio- Rad Laboratories) until bromophenol reached the point of 0.5 cm to the end of the agarose gel. After electrophoresis, the separated skim milk proteins were transferred onto nitrocellulose membrane (Serva Electrophoresis GmbH, Heidelberg, Germany) according to the Towbin method [43] for 1.5 h (250 mA).

### Colloidal silver staining

After transfer of separated glycoproteins onto nitrocellulose, the membrane was washed in deionized water and stained with a colloidal silver staining solution [44], prepared by mixing of 2.5 ml of 40 % sodium citrate dihydrate, 2 ml of 20 % ferrous sulfate heptahydrate (freshly prepared), 0.5 ml of 20 % silver nitrate and 45 ml of deionized water. The colloidal silver staining lasted 10 min and was stopped by adding deionized water. The membrane was dried, scanned, and analyzed. The colloidal silver staining was carried out twice, and the results are presented as the mean value obtained from two experiments.

### Lectin- blotting

The reactivities of fucose-specific lectins with human skim milk glycoprotein bands were analyzed by lectin- blotting using UEA, LCA and LTA biotin-labeled lectins (Vector Laboratories Inc., Burlingame, USA) with well-known specificity (Table 1). The lectin- blotting details are as follows: after the SDS-PAGE, the separated milk proteins as well as positive (human haptoglobin and an asialo-haptoglobin preparation derived from ovarian cancer fluid) [45] and negative (human albumin) controls were transferred onto nitrocellulose membrane, and then the membrane was blocked with 2 % Tween-20 in TBS, pH 7.5 at 32 °C for 1 h. After washing with 0.1 % Tween-20 in TBS (TBS-T) (at 32 °C for 1 × 15 and 2 × 5 min), the membranes were incubated with biotin-labeled lectins: UEA (2 μg/ml), LCA (0.4 μg/ml), and LTA (4 μg/ml) for 1 h at 32 °C in TBS containing 0.1 % Tween 20, 1 mM MgCl_2_, 1 mM CaCl_2_, 1 mM MnCl_2_, pH 7.5. The formed lectin-glycoprotein complex was detected by the reaction with ExtrAvidin phosphatase-labeled (Sigma, St. Louis, MO, USA) diluted 1:20 000 in TBS containing 0.1 % Tween 20, pH 7.5 (blots were incubated at 32 °C for 1 h). After washing with TBS-T, the colored reaction was developed by incubating the nitrocellulose in a freshly prepared solution: 80 μl of BCIP-phosphate (5-bromo-4-chloro-3-indolylphosphate, Sigma, St. Louis, MD, USA) and 80 μl of NBT-chloride (nitroblue tetrazolium chloride, Sigma, St. Louis, MD, USA) in 8 ml of 0.1 M Tris/HCl, pH 9.5, containing 0.05 M MgCl_2_, 0.1 M NaCl at room temperature for 50 s. The blots with all lectins were carried out twice, and the results are presented as the mean value obtained from two experiments.

**Table 1:** Major binding characteristics of some fucose-specific lectins

Origin and used abbreviation of lectin	Binding preferences
*Ulex europaeus* agglutinin (UEA)	Fucose linked α1-2 to Gal of N- and O-glycans [54, 55]
*Lens culinaris* agglutinin (LCA)	Fucose linked α1-6 to proximal GlcNAc of the trimannosyl core of biantennary N-glycans [56]
*Lotus tetragonolobus* agglutinin (LTA)	Fucose linked α1-3 to subterminal GlcNAc of N- and O-glycans [55, 57]

### Densitometric analysis

The intensity of bands obtained after silver staining and lectin- blotting corresponded to the amount of proteins per band and lectin reactivity with the particular fucosylated glycoproteins of skim milk, respectively. The intensity of each line from blots was quantified by densitometric analysis with the myImageAnalysis software (Thermo Scientific, New Hampshire). The relative amount of protein bands and the fucosylated glycotopes on each glycoprotein band was expressed as a mean of pixels × 10^6^ for the individual band obtained from two blots.

### Glycoprotein molecular mass determination

To determine the molecular mass of separated human skim milk glycoproteins, the Precision Plus Protein standards for SDS-PAGE (Bio-Rad) containing 10 protein standards with molecular mass ranging from 250 to 10 kDa were used. Based on molecular mass of standards, a calibration curve of the log of molecular mass (Mm) versus R_f_ was generated.

### Statistical analysis

The statistical analysis was performed with the STATISTICA 10.0 software package (StatSoft, Inc., Tulsa, OK, USA). The correlations were estimated according to Spearman. A *p*-value lower than 0.05 was regarded as significant.

## Results

### SDS-PAGE pattern of human skim milk proteins

**Fig. 1:**
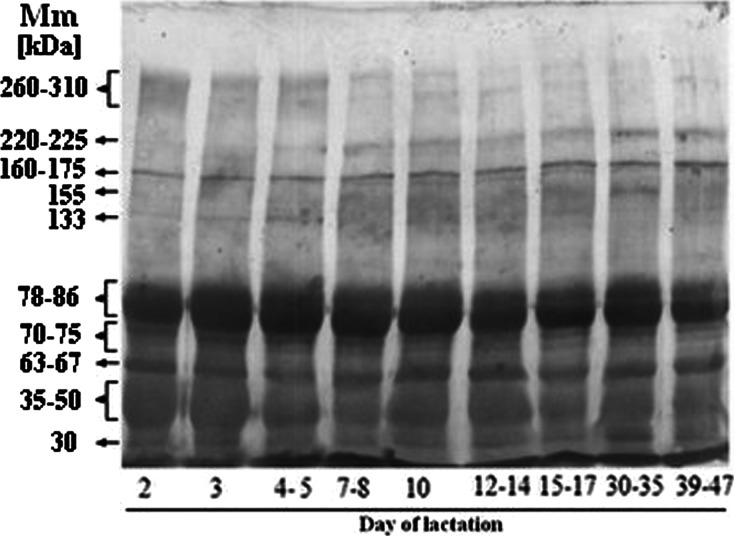
Representative patterns of human skim milk proteins over lactation. A human skim milk sample containing 30 μg of proteins was loaded per lane of SDS-PAGE. After separation the proteins were transferred onto nitrocellulose, and the membrane was stained with a colloidal silver [44]. For experimental details see Material and methods

Among ten milk protein bands revealed by SDS-PAGE and showing molecular masses ranging from 30 to 310 kDa, the most abundant were three: 260–310 kDa (17.7 %), 78–86 kDa (26.1 %) and 35–50 kDa (25.1 %) on the first 3 days of lactation (Fig. 1, Table 2).

**Table 2:** Relative amounts of human skim milk proteins over lactation

Mm of protein band [kDa]	Relative amounts of the protein bands [pixels × 10^6^] shown in Fig. 1									r
	Day of lactation									
	2^nd^	3^rd^	4^th^–5^th^	7^th^–8^th^	10^th^	12^th^–14^th^	15^th^–17^th^	30^th^–35^th^	39^th^–47^th^	
260–310	41.0	32.0	30.0	18.0	18.0	18.0	15.0	8.2	18.0	-0.87
220–225	8.3	10.0	6.7	8.7	10.0	8.1	9.6	13.0	11.0	NS
160–175	9.2	6.8	11.0	9.2	7.6	7.9	9.9	9.0	10.0	NS
155	9.7	11.0	9.6	11.0	13.0	12.0	13.0	8.2	12.0	NS
133	8.4	6.6	8.0	4.8	8.0	6.9	8.6	8.5	7.7	NS
78–86	53.0	59.0	63.0	58.0	57.0	52.0	53.0	58.0	49.0	NS
70–75	18.0	19.0	16.0	17.0	20.0	15.0	16.0	17.0	18.0	NS
63–67	18.0	17.0	14.0	17.0	18.0	18.0	17.0	20.0	16.0	NS
35–50	58.0	57.0	49.0	44.0	45.0	46.0	41.0	43.0	40.0	-0.92
30	8.8	9.4	8.9	11.0	8.7	10.0	8.2	10.0	8.1	NS

The pattern of bands shown in Fig. 1 was analyzed using myImageAnalysis software (Thermo Scientific, New Hampshire)

The relative amount of protein bands was expressed as the mean number of pixels × 10^6^ obtained from two blots

r - correlation coefficient with lactation days

*NS* not significant with a *p*-value equal to or higher than 0.05

Over milk maturation the relative amounts of the 260–310 and 35–50 kDa bands showed a high negative correlation with the day of lactation (r = -0.87 and -0.92, respectively). In contrast, the relative amounts of the 220–225,160–175, 155, 133, 78–86, 70–75, 63–67 and 30 kDa protein bands did not significantly change during the course of lactation (Fig. 1, Table 2).

### Fucose-specific lectin- reactivity with human milk glycoproteins

**Fig. 2:**
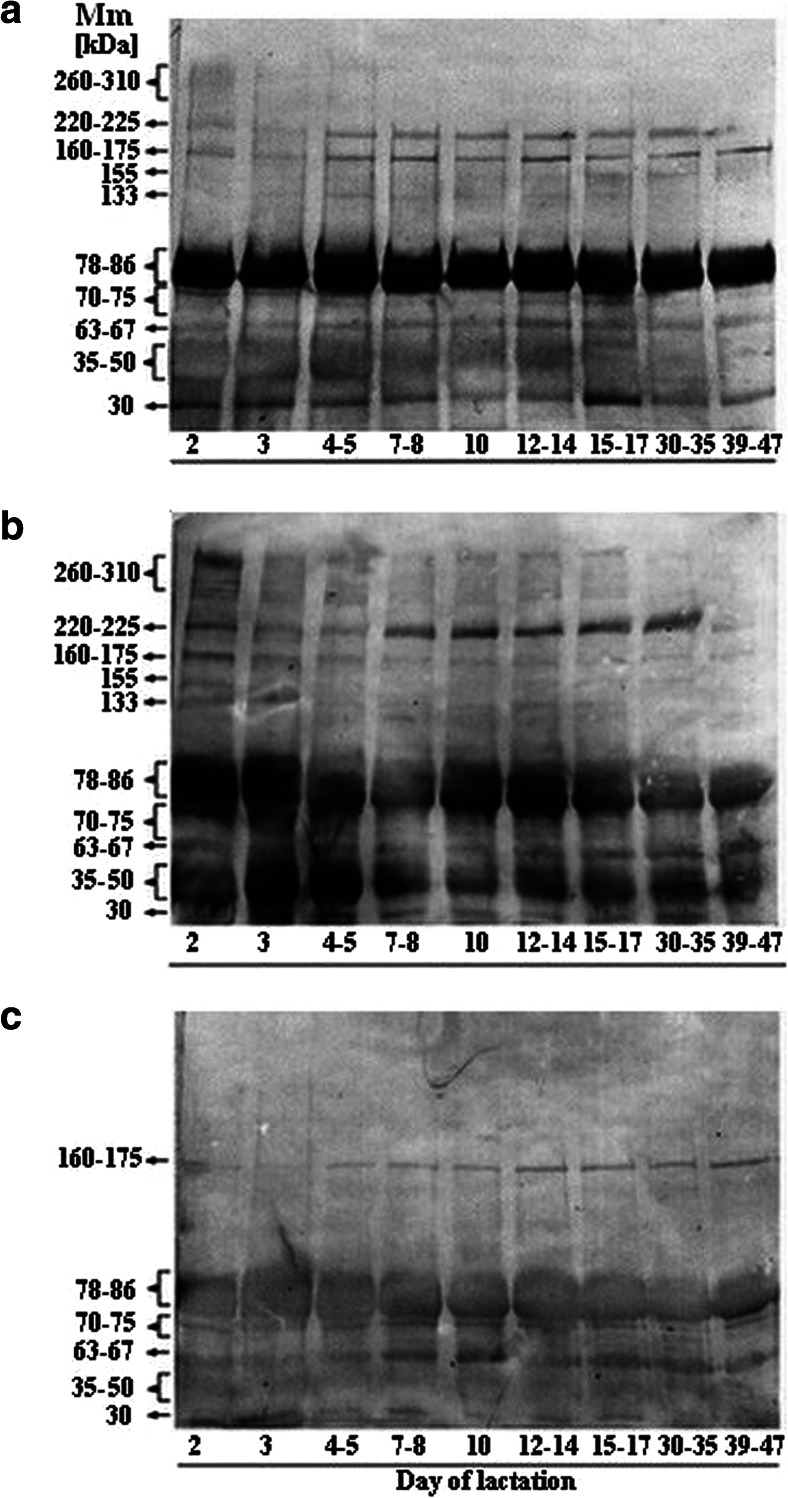
Representative patterns of human milk glycoprotein reactivities with fucose-specific lectins over lactation. For analysis of fucosylation of milk glycoproteins, the samples were collected only from mothers who have secretor status. The pooled milk sample containing 30 μg of proteins was loaded per lane of SDS-PAGE. After separation, the glycoproteins were transferred onto nitrocellulose, and the membrane was subjected to the reaction with (**a**) *Ulex europaeus*, (**b**) *Lens culinaris*, and (**c**) *Lotus tetragonolobus* biotinylated lectin (Vector Laboratories Inc. Burlingame. USA), respectively. The formed lectin-glycoprotein complex was detected by the reaction with phosphatase-labeled ExtrAvidin (Sigma. St. Louis. MO. USA). All blots were done in duplicate. For experimental details see Material and methods

The lectin- blotting pattern of human skim milk glycoproteins over the course of lactation showed strong reactivities with UEA and LCA, but with LTA remained (if at all) at a low level (Fig. 2). The semi-quantitative differences between the particular milk glycoprotein-lectin reactivities over the course of lactation are given in Table 3.

**Table 3:** Relative amounts of α1-2-, α1-3- and α1-6-fucosylated glycotopes on human skim milk glycoproteins over lactation

Mm of protein band [kDa]	Lectin (glycotope recognized)	Relative amounts of the glycotope [pixels × 10^6^] revealed by lectin-immunoblotting shown in Fig. 2									r
		Day of lactation									
		2^nd^	3^rd^	4^th^–5^th^	7^th^–8^th^	10^th^	12^th^–14^th^	15^th^–17^th^	30^th^–35^th^	39^th^–47^th^	
260–310	UEA (α1-2-Fuc)	9.5	0	0	0	0	0	0	0	0	ND
	LCA (α1-6-Fuc)	14.9	5.3	4.2	1.0	1.5	2.1	1.7	0	0	-0.85
	LTA (α1-3-Fuc)	0	0	0	0	0	0	0	0	0	ND
220–225	UEA (α1-2-Fuc)	5.0	3.8	4.6	6.2	4.6	4.1	3.8	3.1	1.2	-0.77
	LCA (α1-6-Fuc)	5.3	4.0	4.5	5.3	6.9	5.3	5.2	5.4	2.6	NS
	LTA (α1-3-Fuc)	0	0	0	0	0	0	0	0	0	ND
160–175	UEA (α1-2-Fuc)	6.6	5.8	6.9	6.9	5.1	6.1	4.4	3.8	4.5	-0.72
	LCA (α1-6-Fuc)	5.3	3.4	3.5	2.3	3.8	2.9	2.1	2.2	2.0	-0.83
	LTA (α1-3-Fuc)	1.6	1.2	1.0	1.1	1.2	1.4	1.5	1.4	1.7	NS
155	UEA (α1-2-Fuc)	0	0	0	0	0	0	3.8	3.4	2.1	ND
	LCA (α1-6-Fuc)	2.4	0	0	0	0	0	0	0	0	ND
	LTA (α1-3-Fuc)	0	0	0	0	0	0	0	0	0	ND
133	UEA (α1-2-Fuc)	2.4	2.7	3.2	1.7	2.6	1.8	3.0	0	0	NS
	LCA (α1-6-Fuc)	2.8	2.8	1.7	1.5	1.8	1.9	0	0	0	-0.81
	LTA (α1-3-Fuc)	0	0	0	0	0	0	0	0	0	ND
78–86	UEA (α1-2-Fuc)	78.7	82.1	80.9	82.4	75.0	83.7	79.8	69.8	69.1	NS
	LCA (α1-6-Fuc)	48.0	45.8	37.3	37.1	41.1	42.1	30.1	26.5	26.0	-0.85
	LTA (α1-3-Fuc)	10.9	11.3	12.9	13.4	12.9	14.0	12.1	11.0	10.6	NS
70–75	UEA (α1-2-Fuc)	15.1	4.3	3.4	2.5	5.9	5.7	6.9	2.7	1.7	NS
	LCA (α1-6-Fuc)	3.2	3.6	3.5	2.1	3.6	3.2	2.5	1.2	0	-0.70
	LTA (α1-3-Fuc)	1.8	1.8	2.0	2.1	2.2	2.1	2.1	1.8	1.8	NS
63–67	UEA (α1-2-Fuc)	11.2	6.8	10.6	9.9	8.6	11.9	11.4	8.1	5.1	NS
	LCA (α1-6-Fuc)	9.8	5.8	7.1	9.2	6.6	6.7	5.6	4.9	6.4	NS
	LTA (α1-3-Fuc)	4.0	2.7	3.5	4.2	4.7	3.5	5.8	4.7	3.9	NS
35–50	UEA (α1-2-Fuc)	29.6	23.5	30.4	23.0	25.2	26.2	16.9	4.3	0	-0.77
	LCA (α1-6-Fuc)	29.0	31.7	23.8	15.0	16.2	18.0	10.0	12.8	11.0	-0.87
	LTA (α1-3-Fuc)	3.1	1.9	1.6	1.7	1.6	2.2	2.8	1.5	1.5	NS
30	UEA (α1-2-Fuc)	12.3	14.7	13.8	9.8	8.8	11.7	14.4	9.6	9.6	NS
	LCA (α1-6-Fuc)	4.1	2.9	2.6	3.0	2.0	1.9	1.9	2.9	1.8	-0.75
	LTA (α1-3-Fuc)	2.0	2.4	1.4	1.8	0.8	1.5	1.6	0.6	0	-0.78

For analysis of fucosylation of milk glycoproteins, the samples were collected only from mothers who have secretor status

The fucosylated bands revealed by lectin- immunoblotting and shown in Fig. 2 were analyzed using myImageAnalysis software (Thermo Scientific, New Hampshire). The relative amounts of fucosyl- glycotopes on each glycoprotein band are expressed as the mean number of pixels × 10^6^ obtained from two independently done blots

r - correlation coefficient with lactation days

*NS* not significant with a *p*-value equal to or higher than 0.05; *ND* not determined

### Expression of UEA-reactive bands

The UEA- reactivity (Fig. 2a, Table 3) of the 220–225, 160–175, and 35–50 kDa glycoproteins showed a strong negative correlation over the progression of lactation (r = -0.77, -0.72 and -0.77, respectively) (Table 3), whereas the reactivity of the 133 kDa, 78–86, 70–75, 63–67, and 30 kDa glycoproteins did not (Table 3). Moreover, the 260–310 and 155 kDa glycoproteins lacked the UEA-reactive glycotopes during the course of lactation, but with time-dependent exceptions, *i.e.*, low UEA- reactivity observed for the colostrum sample from the 2^nd^ day and the mature milk samples from the 15^th^–47^th^ days, respectively.

### Expression of LCA-reactive bands

The LCA- reactivity (Fig. 2b, Table 3) of the 260–310 kDa (r = -0.85), 160–175 kDa (-0.83), 133 kDa (r = -0.81), 78–86 kDa (-0.85), 70–75 kDa (r = -0.70), 35–50 kDa (r = -0.87) and 30 kDa (r = -0.75) glycoprotein bands showed a strong negative correlation over the progression of lactation. In contrast, the relative reactivities of LCA-reactive glycotopes of 220–225 and 63–67 kDa glycoproteins did not significantly change over the progression of lactation. The 155 kDa band was not recognized by LCA over lactation with the exception of low reactivity (1.9 %) observed on the 2^nd^ day of lactation (Table 3).

### Expression of LTA-reactive bands

LTA reacted with 30, 35–50, 63–67, 70–75, 78–86 and 160–175 kDa glycoproteins of human milk (Fig. 2c, Table 3), but only the 30 kDa band showed a negative correlation (r = -0.78) with progression of lactation. In contrast, the 260–310, 220–225, 155 and 133 kDa bands were not recognized by LTA at all (Table 3).

### Pattern of fucose-specific lectin- reactivity with human milk glycoproteins

**Fig. 3:**
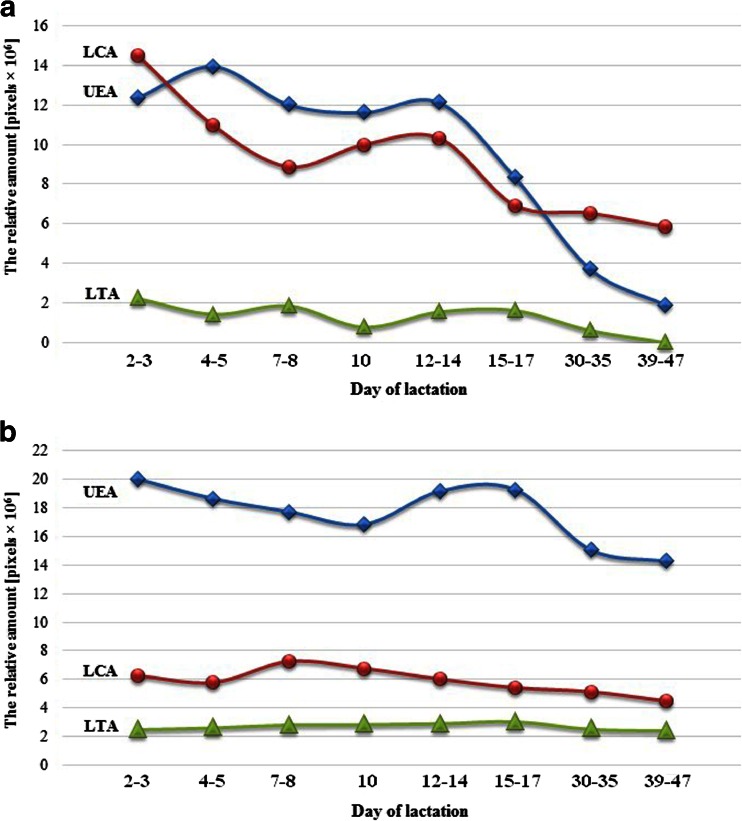
General pattern of fucose-specific lectin- reactivity of human milk glycoproteins over lactation. The mean value of the relative amounts of the lectin-reactive milk glycoproteins was calculated for those showing (**a**) a significant correlation and (**b**) no significant correlation with milk maturation. For the calculation, the following SDS-PAGE bands (Table 3) were selected in: (**a**): reactive with UEA - 220–225 kDa, 160–175 kDa, 35–50 kDa;, LCA - 260–310 kDa, 160–175 kDa, 133 kDa, 78–86 kDa, 70–75 kDa, 35–50 kDa, 30 kDa; and LTA - 30 kDa; and in (**b**): reactive with UEA - 260–310 kDa, 133 kDa, 78–86 kDa, 70–75 kDa, 63–67 kDa, 30 kDa; LCA - 220–225 kDa, 63–67 kDa;, and LTA - 260–310 kDa, 220–225 kDa, 160–175 kDa, 133 kDa, 78–86 kDa, 70–75 kDa, 63–67 kDa, 35–50 kDa. For other details see under Table 3

Based on the analyses of the fucosyl-lectin reactivities of milk glycoproteins over the progression of lactation, two general types of changes were selected (Fig. 3). The first one (Fig. 3a) was associated with a significant decline of reactivities of milk UEA-reactive- (220–225 kDa, 160–175 kDa, 35–50 kDa), LCA-reactive- glycoproteins (260–310 kDa, 160–175 kDa, 133 kDa, 133 kDa, 78–86 kDa, 70–75 kDa, 35–50 kDa, 30 kDa), and only one, the LTA-weakly reactive 30 kDa glycoprotein (Fig. 3a). The UEA- and LCA-reactive glycoproteins, but not LTA-reactive 30-kDa glycoprotein, showed the characteristic decline patterns. However, the level of UEA-reactive glycoproteins from the beginning of lactation (2^nd^-3^rd^ day) to the 12^th^–14^th^ day of lactation was relatively stable, in contrast to LCA-reactive glycoproteins, whose level significantly decreased from 2–3 to 7–8 days and remained almost unchanged until the 12^th^–14^th^ day. During the progression of lactation from the 15^th^–17^th^ days to the 39^th^–47^th^ days the reactivities of UEA and LTA with milk glycoproteins significantly decreased (Fig. 3a).

The second type of changes during the 2^nd^ and 39^th^–47^th^ days of lactation was related to a gradual, but insignificant decrease of UEA- (260–310 kDa, 133 kDa, 78–86 kDa, 70–75 kDa, 63–67 kDa, 30 kDa), and LCA-reactive (220–225 kDa, 63–67 kDa) milk glycoproteins (Fig. 3b), and LTA-weakly reactive at a rather stable low level (160–175 kDa, 78–86 kDa, 70–75 kDa, 63–67 kDa, 35–50 kDa) and non-LTA-reactive (260–310 kDa, 220–225 kDa, 133 kDa) milk glycoproteins (Fig. 3b).

## Discussion

The study shows that despite the individual differences in fucosylation of particular human milk glycoproteins, the gradual decline of highly expressed α1-2- and α1-6-fucosylated glycotopes on human milk glycoproteins over lactation was associated with milk maturation, in contrast to a relatively constant low expression or absence of α1-3-fucosylated glycotope.

Among ten protein bands revealed by SDS-PAGE and silver staining (Fig. 1) nine bands, excluding the 155 kDa band, were evidently fucosylated (Fig. 2). The 155-kDa protein is either not fucosylated or its glycotopes are hidden. The fucosylated glycoproteins were recognized by the lectin reagents *in vitro* (Table 2), and being exposed on milk glycoproteins are ready for specific interactions with endogenous respective lectins, *in vivo*. The milk glycoproteins were heavily decorated with α1-2-linked fucose, to a lesser degree with α1-6-linked fucose, and weakly, if at all, with α1-3-linked fucose. Based on the degree of reactivity of fucose-specific lectins with particular skim milk glycoproteins, two types of general patterns of α1-2-, α1-6-, and α1-3- linked fucose expression over normal lactation might be distinguished (Fig. 3a and b). The first comprises the glycoproteins which showed significant decreases in the α1-2- (3 glycoprotein bands), α1-6- (7 glycoprotein bands), and α1-3-linked fucose (1 glycoprotein band) expression (Fig. 3a) in relation to physiological stages of milk maturation, and the second was variable but did not show a statistically significant correlation (Fig. 3b). Interestingly, a high negative correlation over the progression of milk maturation was shown by about 30, 70, and 10 % of α1-2-, α1-6-, and α1-3-fucosylated glycoproteins, respectively. These glycoproteins showed their highest relative reactivities with UEA, LCA, and LTA in the colostrum at the beginning of lactation (Fig. 3a). Furthermore, over the progression of lactation the profile of fucosyl-glycotope expression differed in relation to the type of glycosidic bond, which links a fucose to the oligosaccharide part of glycoprotein. The high expression of the α1-2-linked fucose remained at a nearly stable level from the beginning of lactation up to the 14^th^ day of lactation, *i.e.*, from colostrums to the transitional milk, and further decreased intensively, reaching about a 6- times lower reactivity value in mature milk. Also, the profile of the α1-6-fucose expression on milk glycoproteins corresponded to milk maturation intervals (Fig. 3a), but its profile differs from that of the α1-2-fucose glycotope. Descending stages happened twice, during the first week and after two weeks of lactation, and they were separated by a week of nearly stable level between 7- and 14 days of lactation. Some small differences in the expression of α1-2- and α1-6-linked fucoses were probably limited by biological lactation variation caused by inherent interindividual differences among the mothers [46].

In contrast, the expression of α1-3-fucose on milk glycoproteins, if present, was not associated with the milk maturation process. Six of ten milk glycoproteins were weakly recognized by LTA and remained at an unchanged level during milk maturation, three of them did not react (Fig. 3a), whereas a low molecular 30-kDa milk glycoprotein reacted evidently, showing a significant negative correlation with the course of lactation. The high expression of α1-3-linked fucose on the colostrum glycoprotein, 2 days after parturition, might reflect the occurrence of Lewis^X^ antigen on 30-kDa milk glycoprotein. Its occurrence was probably associated with the delivery-induced inflammatory state [47] and hormonal changes [48]. During the following days after delivery, the synthesis of inflammation-induced α1-3-fucosylated Lewis^X^ glycotope was reduced together with physiological silencing of the perinatal inflammatory processes.

Interestingly, the glycoproteins which have relatively stable expression of α1-2-linked fucose over the course of lactation are probably lactoferrin, the secretory component of IgA (a wide band of 78–86 kDa), ceruloplasmin, pro-epidermal growth factor (133 kDa), and adiponectin (30 kDa) and are reported to play principal roles in innate and adaptive immunity. The α1-2-fucosylation pattern of 220–225 and 35–50 kDa bands, which may correspond to fibronectin and α_1_-acid glycoprotein, respectively (confirmed by immunoblotting - not shown), overlapped with changes reported previously [38, 39].

Among ten bands revealed by LCA- blotting (Fig. 2), two having 78–86 and 35–50- kDa showed the highest expression of α1-6-fucosylated glycotopes (Table 3). However, over 47 days of lactation the LCA- reactivity of milk glycoproteins significantly decreased and was absent for 260–310, 133, and 70–75 kDa glycoprotein bands.

In spite of the fact that the milk fucosylation pattern can be slightly disturbed by the presence of self-reactive natural blood group antibodies [49] which might precipitate cognate glycoproteins when milk samples were pooled, the characteristic pattern α1-2- and α1-6-fucosylation of glycoproteins seems to be associated with a significant biological role of these glycotopes. Both are needed during the first days of the newborn’s life and are supplied to the breastfed infant. Those decorated by α1-2-linked fucose provide many immunological benefits to the newborn via milk, which include reduced rates of necrotizing enterocolitis, diarrhea, sepsis and urinary tract infections [50]. The α1-2-fucosylated glycotopes of HMOs and glycoproteins can act as ligands for lectin- receptors of bacteria and/or viruses as well as for lectin receptors of newborns’ epithelial cells. The ‘two-way’ participation of human milk glycoproteins in binding and inhibition of pathogen adhesion to the host cells has been confirmed for lactoferrin [51]. Moreover, α1-2-fucosylated glycotopes of human milk glycoproteins, similar to fucosylated HMOs, may take part in improvement of neurocognitive development, promotion of early development of normal bacterial flora in the newborn’s gastrointestinal tract and postnatal maturation of intestinal motor activity in infants [50, 52, 53].

The α1-6-fucosylated glycotopes on milk glycoproteins, which are absent on HMOs [23], may constitute an additional source of the ligand for gastrointestinal tract epithelial lectin receptors and can be considered as a possible modulator of gastrointestinal cell maturation, when the immune system and gastrointestinal tract of the newborn are just beginning to adapt to the new conditions.

The trends observed for major α1-2-fucosylated milk glycoproteins during conversion of immature colostrums throughout transitional to mature milk overlap with those observed for overall fucosylation of HMOs and the levels of 2′ and 3-fucosyllactose [23]. The observed changes in the α1-6- and α1-2-fucosylation of milk glycoproteins during 7 weeks of normal lactation corresponded to physiological stages of milk maturation. In the first weeks of the newborn’s life, the fucosylated glycoproteins given by the mother to the breastfed newborn enrich the immature immune system of the newborn, and they seem to be crucial for the newborn’s needs for proper development and well-being. Therefore, human milk of donors and milk formula, particularly for preterm newborns, should correspond to the fucosylation milk profile of healthy mothers.

## Abbreviations

HMOs: Human Milk Oligosaccharides
LCA: Lectin from *Lens culinaris*
LTA: Lectin from *Lotus tetragonolobus*
UEA: Lectin from *Ulex europaeus*
Fuc: fucose
